# Contemporary Anti-Retroviral Drugs (ARVDs) Disrupt Follicular Development in Female Wistar Rats

**DOI:** 10.2147/JEP.S398343

**Published:** 2023-07-03

**Authors:** Aigbe Gregory Ohihoin, Esther Ngozi Ohihoin, Ifeoma Ujomu, Airat Bakare, Oladeji Olanrewaju, Arinze Okafor, Mercy Ojetunde, Joy Boluwatife Ayoola, Oluwagbemiga Aina, Olusola Ajibaye, Simon D Taylor-Robinson

**Affiliations:** 1 Clinical Sciences Department, Nigerian Institute of Medical Research (NIMR), Lagos, Nigeria; 2 Research Unit, HICI Healthcare Limited, Lagos, Nigeria; 3 University Hospitals of Leicester NHS Trust, Leicester, UK; 4 College of Medicine, University of Lagos, Lagos, Nigeria; 5 School of Life Sciences, University of Nottingham, Nottingham, UK; 6 Central Research Laboratory, Nigerian Institute of Medical Research (NIMR), Lagos, Nigeria; 7 Biochemistry and Nutrition Department, Nigerian Institute of Medical Research (NIMR), Lagos, Nigeria; 8 Department of Surgery and Cancer, Imperial College London, London, UK

**Keywords:** highly active anti-retroviral therapy, HAART, estradiol, anti-Mullerian hormone, human immunodeficiency virus, HIV, Nigeria

## Abstract

**Introduction:**

There are genuine concerns that long-term use of anti-retroviral drugs may be associated with reproductive complications in females. This study aimed to ascertain the effect of highly active anti-retroviral drugs on the ovarian reserve and reproductive potential of female Wistar rats and by extension to HIV-positive human females.

**Methods:**

A total of 25 female Wistar rats, weighing between 140g and 162g, were randomly allotted into non-intervention and intervention groups, receiving the anti-retroviral drugs, Efavirenz (EFV), Tenofovir Disoproxil Fumarate (TDF), Lamivudine (3TC), and a fixed-dose combination (FDC). The dosage was administered orally at 8 am daily for 4 weeks. Serum concentrations of anti-Mullerian hormone (AMH), follicle-stimulating hormone (FSH), luteinising hormone (LH), and estradiol were measured using standard biochemical techniques (ELISA). Follicular counts were made on fixed ovarian tissue from the sacrificed rats.

**Results:**

The mean AMH level for the control group and the EFV, TDF, 3TC, and FDC groups were 11.20, 6.75, 7.30, 8.27, and 6.60 pmol/L, respectively. The EFV and FDC groups had the lowest AMH, compared to the other groups, but there was no statistically significant difference in AMH across the groups. The mean count of antral follicles was significantly lower in the group that received EFV when compared to the other groups. The corpus luteal count was significantly higher in the control group than in the intervention groups.

**Conclusion:**

The study demonstrated a disruption in the reproductive hormones of female Wistar rats receiving anti-retroviral regimens containing EFV. Clinical studies are required to determine if these changes are seen in women receiving EFV-based treatment, as this may compromise reproductive function and predispose them to early menopause.

## Introduction

Patients infected with HIV are known to make remarkable improvements in their clinical condition and quality of life when managed with highly active anti-retroviral therapy (HAART). These drug regimens usually comprise the use of three different antiretroviral drugs with at least two different mechanisms of action. This treatment strategy has been shown to reduce the risk of resistance compared with the use of monotherapy or dual therapy.^1^ Despite the positive factors that have been demonstrated from the use of HAART, there are genuine concerns that the long-term usage of these drugs to manage HIV may be associated with some complications and side effects. There is evidence in in vitro and in vivo studies to show that zidovudine and other nucleoside analogues are associated with damage to the gametocytes.^2^ HAART may compromise fertility through the depletion of mitochondrial DNA by inhibition of DNA polymerase.^3^ This mechanism is also seen in the development of metabolic syndrome among individuals who are taking HAART. The use of HAART in HIV-positive women has been shown to be associated with a reduction in the mitochondrial content of the oocytes of these patients.^4^ The metabolic syndrome is associated with polycystic ovarian syndrome and hormonal imbalance, being a factor for a possible increase in cases of sub-fertility associated with patients receiving HAART. Despite the use of HAART, HIV has the potential to proliferate in certain anatomical and tissue spaces. This ability contributes to the development of the persistence of the virus, complications, and the emergence of anti-retroviral drug resistance.^5^ Apart from the female genital tract, other anatomic structures exist as reservoirs, this includes the gastrointestinal tract, brain lungs, and the lymphoid tissues.^6^ The use of HAART is also associated with the development of oxidative stress.^7^

The ovarian reserve is a measure of the ovarian store and reflects the quantity and quality of oocytes available for ovulation, essentially a test of fertility potential and a reflection of follicular development.^8^ The American College of Obstetricians and Gynecologists (ACOG) recommends that anti-Mullerian hormone (AMH) estimation and antral follicle count are two reliable methods of determination of ovarian reserve.^8^ With the advancement in reproductive age, there is diminished ovarian reserve. There is a marginal decline in the ovarian reserve of females above the age of 30 years and a significant decline in the ovarian reserve above the age of 40 years.^9^ There has been concern about the development of anovulation and early menopause in patients living with HIV, but there is no certainty as to whether this is related to the progress of the disease or the use of HAART.^10^

In HIV-positive individuals being evaluated for infertility, there is some evidence to suggest that they show a diminished ovarian reserve, as demonstrated by AMH levels when compared with matched controls of HIV-negative women who are also being evaluated for infertility.^11^ It is not clear if diminished AMH levels can be ascribed to the disease or HAART. We, therefore, aimed to determine this further in a rat model, and specifically, to demonstrate the effect of first-line HAART on the follicular development of female Wistar rats, as ascertained by AMH levels. The rationale for conducting this study in an animal model was also to ensure a detailed study of the morphology and histology of ovarian tissue. We studied five groups of female rats – a control group, one group each receiving efavirenz (EFV), tenofovir (TDF) and lamivudine (3TC) and to assess drug interaction, a fifth group that received a fixed dose combination of all three drugs.

## Methods

The host institution, the Nigerian Institute of Medical Research (NIMR), conducts animal research in scientific laboratories in accordance with the Basel Declaration on animal research standards and those outlined by the International Council for Laboratory Animal Science (ICLAS).

Prior ethics approval for the study was obtained in accordance with these precepts from the local research ethics committee (LREC) of NIMR in Lagos, Nigeria (NIMR REC references 2018/0201 and A0724/42). As set out in the ethical approval process, the study complied with the United Kingdom revised Animals (Scientific Procedures) Act 1996 and the European Union Directive 2010/63/EU. The ethics committee was mindful of the 3Rs in animal research (replacement, reduction and refinement) and only gave their approval on study design when sample size had been justified for the minimum number of animals to be used to achieve the study aims.

A total of 25 female Wistar rats were weighed using an animal weighing balance (Kent Scientific Inc., Torrington, CT, USA). The animals weighed between 140g and 162g. The age of the animals ranged from 12 weeks to 20 weeks. They were kept in the experimental animal house of the Yaba Campus of NIMR in Lagos, Nigeria. The animals were housed in clean, steel cages at a temperature of 28°C.

The animals were kept in accordance with conditions stipulated by the US National Institutes of Health (NIH), involving a cycle of 12 hours light and dark, being the institutional ethics committee-enforced standard required for the care of laboratory animals.

The animals were fed standardized laboratory chow (Livestock Feeds Ltd, Ikeja, Nigeria) and were allowed 2 weeks to acclimatize to their new environment. The animals were then observed for a further 4 weeks during the dosing study, receiving daily treatment at 8 am through an oral cannula, having been allotted randomly into 5 groups with five animals per group.

### Dosing Pattern and Procedure for Drug Administration

The dosing pattern was based on per kg body weight of the usage of the drugs in humans and extrapolated to per kg body weight usage of the drugs in the Wistar rat model, as advised by the pharmaceutical manufacturers. Water that was administered was given 0.2mL across animals irrespective of weight with the use of a 1mL hypodermic syringe.

#### Group 1 (Control)

This group was given water as a placebo at a dose of 0.2mL (five animals).

The experimental animals in the other four groups received the anti-retroviral drugs: Efavirenz (EFV), Tenofovir (TDF) and Lamivudine (3TC) at measured doses for weight for a period of 4 weeks.

#### Group 2: Efavirenz

This group received EFV. The brand used was Strides 200mg film-coated oral tablets (Strides Pharma Science Limited, Bengaluru, India). EFV has very low aqueous solubility (9.19 microgram/mL). Half a tablet was dissolved in 100mL of distilled water to form a 1mg/mL solution. The 200mg tablet, which is scored, was split with a tablet cutter. The half tablet was crushed in a porcelain mortar and rinsed into a conical flask. The administered dose was 50mg/kg (2.5mg/50g body weight). Thus, 2.5mL of 1mg/mL solution was given to a rat weighing approximately 150g. The solution was shaken very well before withdrawal with a syringe to ensure uniformity of dose.

#### Group 3: Tenofovir

This group was given TDF. The brand used was Mylan 300mg film-coated oral tablet (Mylan NV, Canonsburg, PA, USA). The solubility was 13.4mg/mL. A quarter (1/4) tablet (75mg) was dissolved in 100mL of distilled water to obtain 0.75mg/mL solution. The 300mg tablet which was scored was split with a tablet cutter; the resulting half tablet was again split to form a quarter tablet. The quarter tablet was crushed in a porcelain mortar and rinsed into a conical flask. The administered dose was 17mg/kg (0.85mg/50g). About 1.1mL of 0.17mg/mL solution was given to a 150g rat. The solution was shaken very well before withdrawal with a syringe to ensure uniformity of dose.

#### Group 4: Lamivudine

This group was given Lamivudine (3TC). The brand used was Mylan in a 150mg film-coated oral tablet (Mylan NV, Canonsburg, PA, USA). The predicted solubility of lamivudine was 2.76mg/mL which was approximately 3mg. Therefore, one tablet was dissolved in 100mL of distilled water to form a 0.3mg/mL of solution. The tablet was crushed in a porcelain mortar and rinsed into a conical flask. A 2mL oral syringe was used to administer the appropriate dose to the rat. The administered dose was 4mg/kg (0.2mg/50g body weight). Thus, 0.75mL of 0.3mg/mL of solution was administered to a rat weighing approximately 150 g.

#### Group 5: Fixed-Dose Combination (FDC)

This group received a combination of EFV, TDF, and 3TC as a fixed-dose combination. The dose combination comprised EFV at 50mg/kg body weight, TDF at 17mg/kg body weight, and 3TC at 4mg/kg body weight in a fixed-dose combination.

#### Vaginal Fluid Examination

The daily procedure involved the use of a dropper that was rinsed in distilled water and filled with 10μL of normal saline. The dropper with normal saline was carefully inserted into the vagina of the rats to express the vaginal fluid daily to determine what phase of the cycle the rat was passing through.

The vaginal fluid was subsequently placed on a slide for examination under a light microscope using x40 magnification (Olympus Microscopes, Shinjuku, Tokyo, Japan). The smears were evaluated for the characteristic cell type and used for the identification of the various phases of the estrous cycle. The dominance of leukocytes in the smear was indicative of the diestrus phase, while the dominance of nucleated epithelial cells was taken to indicate proestrus. The presence of cornified epithelial cells along with some cell debris in the smear was taken to indicate estrus. The metestrus was identified by the presence of mixed leucocyte and cornified cells.

After anaesthesia with the inhalational anaesthetic, isoflurane, in accordance with American Veterinary Medical Association Guidelines, the rats were sacrificed by cervical dislocation at the end of the 4 weeks. The animals were sacrificed during the estrus phase of the cycle. Blood samples were obtained through cardiac puncture.

The ovaries, oviducts, and the uterus were harvested for analysis. These organs were initially placed in saline solution because the saline solution was considered good at maintaining the tissue parenchyma of lower animals, such as rats (without disrupting the tissue structural integrity), before transferring to formalin solution.

Serum concentrations of follicle-stimulating hormone (FSH), luteinizing hormone (LH), estradiol, and AMH were determined using ELISA kits (Biocode Ltd, Brussels, Belgium).

### Histology and Follicular Count

The ovarian tissue obtained was fixed in 10% formalin and embedded in paraffin wax. To study folliculogenesis, all tissue blocks were serially sliced. Follicle identification was based on the detection of a nucleus. The numbers of follicles were counted. Follicle recognition criteria were based on the type of epithelial cells surrounding them. For example, primordial follicles were attributed if they were surrounded by a single layer of flattened granulosa cells, whereas primary follicles were attributed if they were surrounded by single or several layers of cuboidal granulosa cells.

This process was followed by an examination under the light microscope at a magnification of x100 (Olympus Microscopes, Shinjuku, Tokyo, Japan). Micrographs were taken.^12^

### Statistical Analysis

The size of each study group was calculated using Mead’s resource equation:

$E = N- B- T$, where

- N was the total number of individuals in the study (minus 1) = 24.
- *B* was the blocking component, representing environmental effects allowed for in the design (minus 1) = 0.
- *T* was the treatment component, corresponding to the number of groups being used, or the number of questions being asked (minus 1) = 4.
- *E* is the degrees of freedom of the error component and should be somewhere between 10 and 20 = 19 in this study, based on 5 groups of 5 rats.

The statistical package for social sciences (SPSS) version 22 was used for statistical analysis (SPSS, Mountain View, California, USA). The results obtained were expressed using mean values ± SD. Data were tested for normality using the Shapiro–Wilk test. Statistical significance was then evaluated via a one-way analysis of variance (ANOVA) and the Student’s *t*-test. P < 0.05 were considered statistically significant. Statistical advice was obtained at study design stage from the Ethics Committee at the Nigerian Institute of Medical Research in Lagos, Nigeria. The results of the study are reported in accordance with ARRIVE guidelines (arriveguidelines.org).

## Results

The weight of the rats used in the study before the commencement of the intervention ranged between 140 and 160 g with an average of 153g ± 8.78. After the intervention, the weight of the rats had a range of 140–162 g with an average of 153.2g ± 6.91. Weights did not vary before or after the administration of the intervention (p = 0.99).

The mean serum AMH level for the control group and the respective treatment groups of EFV, TDF, 3TC, and FDC were 11.20 ± 1.09, 6.75 ± 0.80, 7.30 ± 1.6, 8.27 ± 0.64, and 6.60 ± 1.44 pmol/L, respectively (p = 0.180) (Table 1). The mean serum estradiol levels for the control group, EFV, TDF, 3TC, and FDC were 10.35 ± 1.73, 9.40 ± 2.15, 27.10 ± 2.04, 15.20 ± 1.78, 17.60 ± 2.18 pmol/L, respectively (p = 0.373) (Table 1). The mean serum LH level for the control group and the respective treatment groups: EFV, TDF, 3TC, and FDC were 0.0420 ± 0.004, 0.0280 ± 0.0018, 0.0320 ± 0.0016, 0.030 ± 0.0018, 0.0320 ± 0.0016 pmol/L, respectively (p = 0.190). Details as shown in Table 1.

**Table 1:** Comparison of Hormone Profile Values Across Group

Hormones	Groups	Average Value (pmol/L)	SD
1. AMH	CONTROL	11.2	1.09
	EFV	6.75	0.8
	TDF	7.3	1.6
	3TC	8.27	0.64
	FDC	6.6	1.44
2. ESTRADIOL	CONTROL	10.35	1.73
	EFV	9.4	2.15
	TDF	27.1	2.04
	3TC	15.2	1.78
	FDC	17.6	2.18
3. LH	CONTROL	0.042	0.004
	EFV	0.028	0.0018
	TDF	0.032	0.0016
	3TC	0.03	0.0018
	FDC	0.032	0.0016
4. FSH	CONTROL	0.92	0.055
	EFV	1.16	0.11
	TDF	0.24	0.014
	3TC	3.16	0.12
	FDC	3.44	0.13

The mean serum FSH levels for the control group and the respective treatment groups of EFV, TDF, 3TC, and FDC were 0.92 ±0.055, 1.16 ± 0.11, 0.24 ± 0.014, 3.16 ± 0.12, and 3.44 pmol/L, respectively (p = 0.224). However, the mean FSH level in the group that received the TDF was 0.238 pmol/L, while that of the control was 0.92 pmol/L. FSH levels were markedly elevated in the group that received the fixed-dose combination of the drugs when compared to the control group. The mean FSH value for the group that received the fixed-dose combination of the drugs was 3.44 pmol/L while that of the control group was 0.92 pmol/L (p = 0.05) (Table 1). In other study groups that received EFV, FSH was marginally elevated when compared to the control group. FSH was, however, elevated in the 3TC group when compared to the control (p = 0.08) (Table 1).

**Figure 1:**
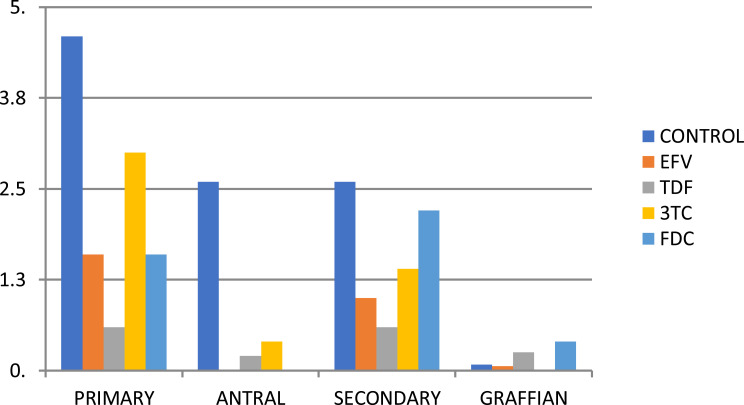
This figure shows a comparison of the follicles in various stages of development for the control (n = 5, in dark blue) and study groups.

**Abbreviations**: EFV, efavirenz group (n = 5, in Orange); TDF, tenofovir group (n = 5, in grey); 3TC, lamivudine group (n = 5 in yellow); FDC, fixed dose combination group (n = 5, in light blue).

The mean count of primary, antral, and secondary follicles was lower in the treatment groups than in the control group. The pattern is shown in the bar chart in Figure 1. The data are shown in tabular format in Table 2. The mean count of primary follicles for the control group and the respective treatment groups of EFV, TDF, 3TC, and FDC were 4.60 ± 0.27, 1.60 ± 0.22, 0.60 ± 0.08, 3.00 ± 0.158, and 1.60 ± 0.19, respectively (Table 2). There was no statistically significant difference across the groups (p = 0.055). The mean count of secondary follicles for the control group and the respective treatment groups of EFV, TDF, 3TC, and FDC were 2.60 ± 0.17, 1.00 ± 0.16, 0.60 ± 0.19, 1.40 ± 0.20, and 2.0 ± 0.14, respectively (p = 0.098) (Table 2). The mean count of antral follicles for the control group and the respective treatment groups of EFV, TDF, 3TC, and FDC were 2.60 ± 0.16, 0.00, 0.20 ± 0.0015, 0.40 ± 0.048, and 0.00, respectively (Table 2). The mean count of antral follicles was significantly lower in the group that received EFV when compared to other groups (p < 0.005). The pattern is shown in the bar chart in Figure 1. The mean corpus luteal count for the control group and the respective treatment groups of EFV, TDF, 3TC, and FDC were 8.00 ± 1.41, 4.40 ± 1.33, 4.00 ± 1.18, 4.80 ± 1.20, and 5.40 ± 1.8, respectively. The corpus luteal count was significantly higher in the control group than in the treatment groups (p = 0.033) (Table 2).

**Table 2:** Comparison of Average Follicular Count

Variable	Control	EFV	TDF	3TC	FDC	P-value
Primary	4.60	1.60	0.60	3.00	1.60	0.055
Antral	2.60	0.00	0.20	0.40	0.00	0.000
Secondary	2.60	1.00	0.60	1.40	2.20	0.098
Graffian	0.08	0.06	0.25	0.00	0.40	0.374
Corpus luteum	8.00	4.40	4.00	4.80	5.40	0.033
AMH	11.2000	6.7500	7.3200	8.2700	6.6000	0.180
Estradiol	10.3500	9.4000	27.1040	15.2000	17.6000	0.373
LH	0.0420	0.0280	0.0320	0.0300	0.0320	0.190
FSH	0.9200	1.1680	0.2380	3.1600	3.4400	0.224

**Notes**: This shows the comparison of average follicular counts in the treatment and control groups (n = 5 animals in each of the five groups), together with serum anti-Mullerian hormone (AMH) levels, serum estradiol levels, serum luteinizing hormone (LH) levels and serum follicle-stimulating hormone (FSH) levels between the treatment groups and the control groups.

**Abbreviations**: EFV, Efavirenz; TDF, Tenofovir; 3TC, Lamivudine; FDC, fixed dose combination of Efavirenz; Tenofovir and Lamivudine.

**Figure 2:**
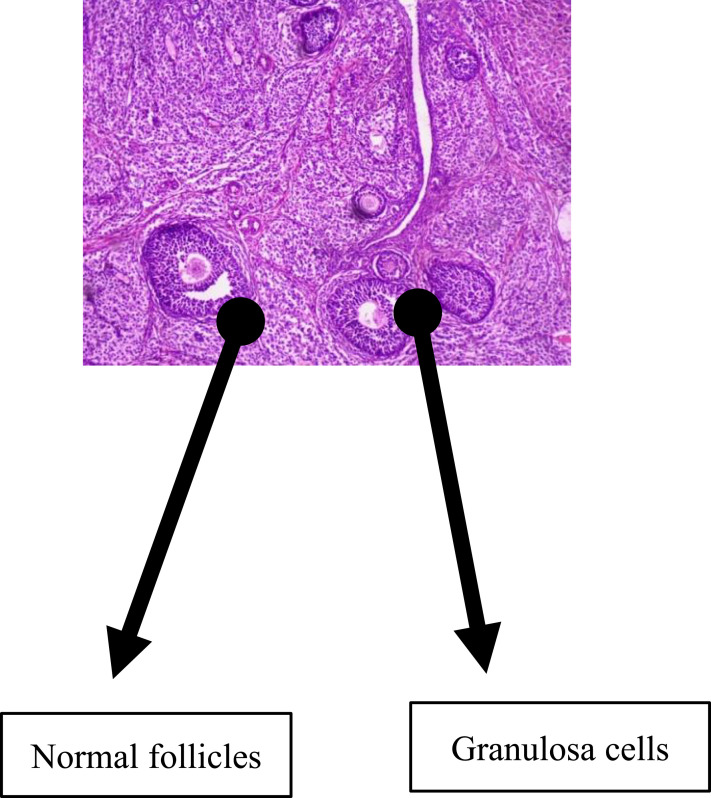
Normal follicular development, with follicles at various stages of development. There are also granulosa cells and basal cells in the control group (group 1) given water as a placebo.

**Figure 3:**
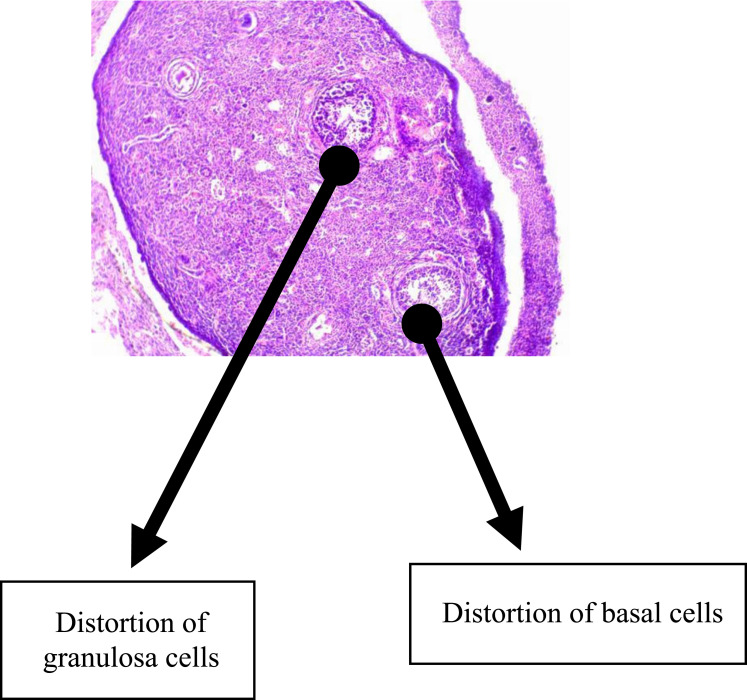
This figure represents the histological findings of the group that received efavirenz, which revealed a distortion of the granulosa and basal cells with a marked reduction of all follicles.

Normal follicular development was demonstrable in the group that received water as a placebo with follicles in this group seen at various stages of development (this is as shown in Figure 2). There are also granulosa cells and basal cells in this control group (group 1), as shown in Figure 2. In the group that received EFV, there was a distortion of the granulosa and basal cells with a marked reduction of all follicles as shown in Figure 3.

**Figure 4:**
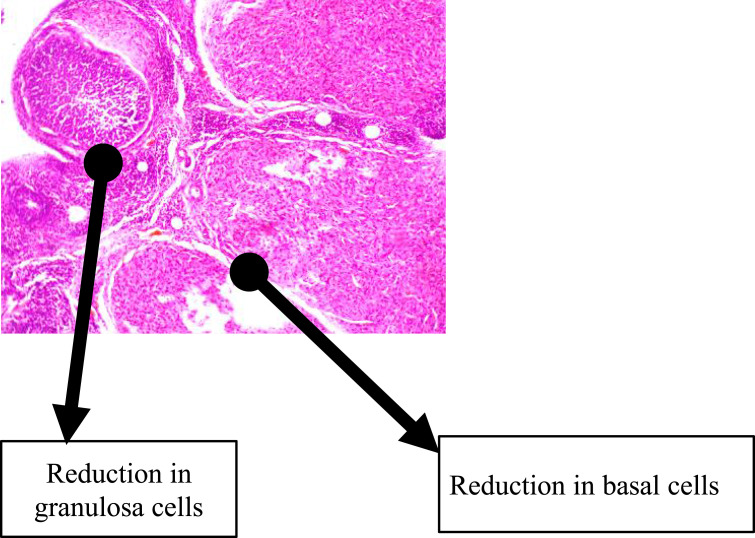
This figure shows the histological findings of the group that received tenofovir, which shows that** **there is a slight reduction in the granulosa and basal cells; follicles at various stages of maturation are distributed over the field.

**Figure 5:**
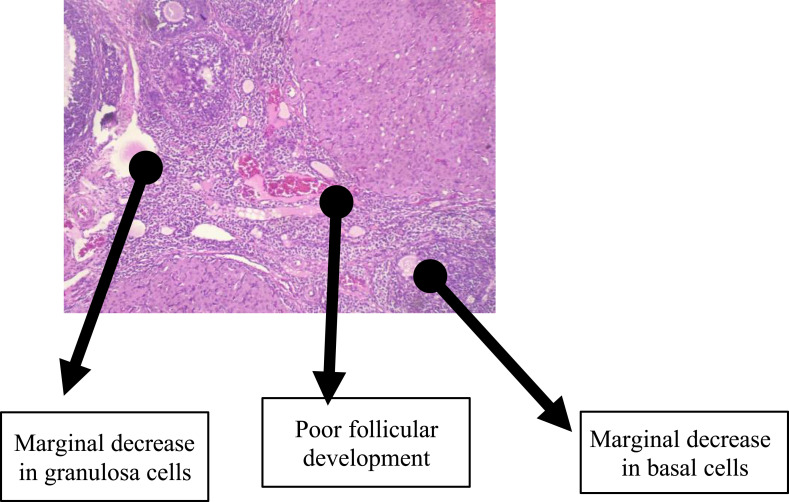
This slide shows the group that received lamivudine which resulted in a marginal decrease in granulosa and basal cells with a less than normal distribution of follicles across various stages of maturation.

**Figure 6:**
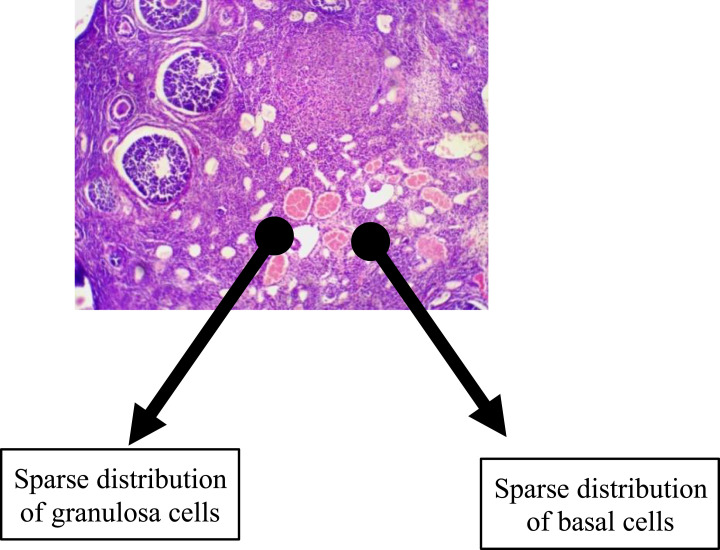
This figure shows the group that received a combination of efavirenz, tenofovir, and lamivudine as a fixed-dose combination had granulosa and basal cells sparsely distributed. The follicles were markedly reduced across various stages of development.

The histological findings of the group that received TDF revealed a non-significant reduction in the granulosa and basal cells with follicles at various stages of maturation distributed over as shown in Figure 4. The administration of 3TC resulted in a marginal decrease in granulosa and basal cells with a less than normal distribution of follicles across various stages of maturation as shown in Figure 5. A representative slide from the FDC group that received a combination of EFV, TDF, and 3TC (as a fixed-dose combination) as shown in Figure 6 had granulosa and basal cells sparsely distributed. The follicles were markedly reduced across various stages of development.

## Discussion

Folliculogenesis is a key aspect of female reproductive function. Disruption of this process leads to compromise in female fertility potential. During folliculogenesis, several follicles are destroyed and end up as atretic follicles.^13^ This study made use of the rat model to study folliculogenesis, owing to the relatively short cycle length averaging 4.8 days.^14^

Findings from the histological analysis of the ovarian tissue of the rats used in this study revealed a reduction in the number of granulosa and basal cells of follicles across various stages of follicular development in the study groups when compared to the control group. This reduction was more marked in the group that received efavirenz. Previous studies on the effect of HAART on reproductive functions have shown highly heterogeneous outcomes. While most of the studies have outlined the effect of antiretroviral medications on male reproductive functions, only a few have looked at the effect of anti-retroviral medications on female reproductive functions. In the study conducted by Awodele et al on rodents to demonstrate the effect of HAART on the reproductive functions of rodents, there was a reduction in follicular development as well as the levels of progesterone, prolactin, and estradiol in the female rodents that received HAART when compared to controls.^15^ This is consistent with findings from our study. However, the exact mechanism of this action has not been elucidated.

Collazos et al studied the effect of HAART on the reproductive hormones of women living with HIV and showed that there was an increase in serum levels of estradiol and testosterone in patients receiving HAART, but levels of LH and FSH were not influenced.^16^ In our study, there was no significant change in the serum levels of LH, while the serum levels of FSH were elevated in the study group that had EFV in the combination. This elevation was statistically significant. While Collazos’ work was on human participants, the present study was conducted in a rat model and this could account for the difference in findings. Additionally, Collazos did not take into account follicular development in his study.^16^

Collazos et al demonstrated that the use of antiretroviral treatment was the only predictive variable for sexual dysfunction in a group of HIV-positive patients managed with antiretroviral therapy.^16^ The authors further stated, however, that sexual dysfunction was not related to hormonal factors in that study.^16^

In our study, serum levels of LH did not show any significant difference between the study groups and the control group. The analysis of the levels of FSH and LH between the study groups and the control revealed a significant degree of variation. FSH levels in the group that received TDF were reduced when compared to the control group. FSH levels were markedly elevated in the group that received the fixed-dose combination of the drugs when compared to the control group. This difference was statistically significant. An elevated level of FSH is a significant marker of menopause as females in menopause demonstrate high levels of FSH due to a marked reduction in follicular development and a reduction in the level of estradiol. The implication of this is that there is no negative feedback of estrogen on FSH levels, hence the marked elevation in the level of FSH itself. The group that received the FDC containing EFV possibly demonstrated elevated levels of FSH due to the toxic effect of the medication on the ovaries, hence follicular damage giving rise to the unopposed action of estrogen and FSH elevation. Awodele et al demonstrated similar findings.^15^

In our other treatment groups that received EFV, FSH was marginally elevated when compared to the control group. FSH was, however, elevated in the 3TC group when compared against controls, but this difference was not statistically significant. FSH levels are known to correlate with ovarian failure and serum levels of estrogen. This relationship is inverse, as depletion of ovarian follicles leads to reduced levels of estrogen.

The group that received 3TC showed a marginal decrease in granulosa and basal cells with a less than normal distribution of follicles across various stages of maturation. The reduction in the number of follicles was more marked in the study group that received EFV when compared to the study groups that received TDF, 3TC, and a combination of all three drugs. The group that received a combination of EFV, TDF, and 3TC as a fixed-dose combination had granulosa and basal cells sparsely distributed. The follicles were markedly reduced across various stages of development. The effect was seen more in follicles at the antral stage of development. A decreased follicular count has been documented in female Wistar rats exposed to *Heracleum persicum*.^17^ This herbal medicinal plant is famously used in Iran for a variety of traditional medicinal purposes. This plant has been used in some traditional settings as a form of contraception. However, this is the first time that such changes have been observed in the context of HAART in this animal model.

AMH is a marker for reproductive potential and ovarian function and also a surrogate for follicular development. It is used for the estimation of ovarian reserve and the prediction of fertility potential. The correlation of AMH with the antral follicular count is because AMH is secreted by the granulosa cells of the developing follicle. As menopause is being approached, AMH values correlate with the number of antral follicles in the ovary. This has been demonstrated in mice as the antral follicular count is directly proportional to the levels of AMH.^18^ Changes in the level of AMH are known to occur relatively early as it relates to ovarian aging.

Furthermore, changes in AMH are known to predate changes in FSH levels and inhibin B.^19^ Thus, AMH seems to be a better predictor of ovarian reserve than Inhibin B and FSH. It also seems a more reliable marker of ovarian reserve, because it does not fluctuate throughout the menstrual cycle.^19^ Of note, the average AMH level in this study was lower in the study groups that received HAART with the combined antiretroviral therapy and EFV alone when compared to the control group, although the difference was not statistically significant. Recent studies tend to suggest that AMH could be protective against ovarian depletion induced by chemotherapy for the treatment of malignancies.^20^

Serum levels of estradiol fluctuate throughout the estrous cycle and as such, estradiol is not a reliable predictor of reproductive function.^19^ Estradiol is produced by the granulosa and theca cells of the developing follicle. The production of estradiol is under the control of gonadotropins from the anterior pituitary. Estradiol regulates female reproductive functions, and it is diminished with aging and depletion of follicles.^13^ HAART has been shown to counteract the contraceptive benefits of the estrogen-containing oral contraceptive pill and invariably leads to a higher contraceptive failure rate.^19^

In our study, there was an increased average level of estradiol in the HAART study groups, when compared to the control group. This change was not statistically significant. However, some workers have noted elevated levels of estradiol in males who are being treated with anti-retroviral drugs; this elevation leads to sexual dysfunction.^20^,^21^ There is a need to further define if this finding applies to HIV-positive women on HAART.

Reduced levels of estrogen obliterate the negative feedback of estrogen on FSH. This leads to a marked elevation of FSH. Elevation of FSH is used as a marker of ovarian function, although it is not as reliable and accurate as the use of AMH and antral follicular count. Concerns have been raised about the age of menopause of women being managed for HIV. It is thought that women who are being managed for HIV will get to menopause earlier than their HIV-negative counterparts.^22^ FSH levels in these individuals have been used as a marker for ovarian function in this group of individuals, although challenges do exist with its usage, due to fluctuation through the menstrual cycle.^23^

## Conclusion

The study demonstrated a disruption in the reproductive hormones of female Wistar rats receiving anti-retroviral regimens containing EFV. Apart from the disruption in these hormones, there is an impairment of follicular development. The study did not interrogate the mechanisms for this abnormality and its implication on the reproductive health of HIV-positive women. There is the need therefore to determine if these changes are seen in women receiving EFV-based treatment, as this may compromise reproductive function and predispose them to early menopause.^24^

## Abbreviations

3TC, Lamivudine; ACOG, American College of Obstetricians and Gynecologists; AMH, anti-Mullerian hormone; EFV, Efavirenz; FDC, fixed-dose combination; FSH, follicle stimulating hormone; HAART, highly active anti-retroviral therapy; IRB, Institutional Review Board; LH, luteinising hormone; NIMR, Nigerian Institute of Medical Research; TDF, Tenofovir Disoproxil Fumarate.

## Data Sharing Statement

Supporting data are available on request from the first author, Dr Aigbe Gregory Ohihoin, Clinical Sciences Department, Nigerian Institute of Medical Research (NIMR), Yaba, Lagos, Nigeria; email aigbe.ohihoin@yahoo.com.

## References

[1] FMOH. Integrated national guidelines for HIV prevention treatment and care; 2016. Available from: http://www.health.gov.ng/. Accessed June 28, 2023.

[2] Brinkman K, Kakuda T. Mitochondrial toxicity of nucleoside analogue reverse transcriptase inhibitors: a looming obstacle for long-term antiretroviral therapy? *Curr Opin Infect Dis*. 2000;13:5–11. doi:10.1097/00001432-200002000-00002 11964766

[3] Lewis W, Dalakas M. Mitochondrial toxicity of antiviral drugs. *Nat Med*. 1995;1:417–422. doi:10.1038/nm0595-417 7585087

[4] López S, Coll O, Durban M, et al. Mitochondrial DNA depletion in oocytes of HIV-infected antiretroviral-treated infertile women. *Antivir Ther*. 2008;13(6):833–838. doi:10.1177/135965350801300607 18839784

[5] Saksena NK, Wang B, Zhou L, Soedjono M, Ho YS, Conceicao V. HIV reservoirs in vivo and new strategies for possible eradication of HIV from the reservoir sites. *HIV/AIDS Res Palliat Care*. 2010;103–122. doi:10.2147/HIV.S6882 PMC3218690 22096389

[6] Poss M, Martin HL, Kreiss JK, et al. Diversity in virus populations from genital secretions and peripheral blood from women recently infected with human immunodeficiency virus type 1. *J Virol*. 1995;69(12):8118–8122. doi:10.1128/jvi.69.12.8118-8122.1995 7494333 PMC189765

[7] Lewis W, Copeland W, Mitochondrial DB. DNA depletion, oxidative stress, and mutation: mechanisms of nucleoside reverse transcriptase inhibitor toxicity. *Lab Invest*. 2001;81:777–790. doi:10.1038/labinvest.3780288 11406640

[8] American College of Obstetrics and Gynecology. Ovarian reserve testing- committee opinion; 2015. Available from: https://www.acog.org. Accessed March 21, 2017. 10.1097/01.AOG.0000459864.68372.ec 25560143

[9] Seifer D, Golub E, Lambert-Messerlian G, et al. Biologic markers of ovarian reserve and reproductive aging: application in a cohort study of HIV infection in women. *Fertil Steril*. 2007;88:1645–1652. doi:10.1016/j.fertnstert.2007.01.122 17418155 PMC2682326

[10] Clark R, Mulligan K, Stamenovic E, et al. Frequency of anovulation and early menopause among women enrolled in selected adult AIDS clinical trials group studies. *J Infect Dis*. 2001;184:1325–1327. 11679923 10.1086/323999

[11] Santulli P, De Villardi D, Gayet V, et al. Decreased ovarian reserve in HIV infected women. *AIDS*. 2016;30:1083–1088. doi:10.1097/QAD.0000000000001025 27028143

[12] Bancroft J, Stevens A. *Theory and Practice of Histological Techniques*. Edinburgh: Churchill Livingstone; 1982.

[13] White B, Porterfield S. *Endocrine and Reproductive Physiology*. 4th ed. Philadelphia, PA: Elsevier/Mosby; 2012.

[14] Andrews WW, Ojeda SRA. Detailed analysis of the serum LH secretory profiles of conscious free-moving female rats during the time of puberty. *Endocrinology*. 1981;109(6):20323–20329. 10.1210/endo-109-6-2032 7198028

[15] Awodele O, Popoola TD, Idowu O, Bashua BM, Awolola NA. Investigations into the risk of reproductive toxicity following exposure to highly active anti-retroviral drugs in Rodents Tokai. *J Exp Clin Med*. 2018;43(2):54–63. 29961933

[16] Collazos J, Martinez E, Mayo J, Ibarra S. Sexual hormones in HIV infected patients: the influence of antiretroviral therapy. *AIDS*. 2002;16(6):934–937. PMID: 11919500. doi:10.1097/00002030-200204120-00018 11919500

[17] Hemati A, Azarnia M, Nabiuni M, et al. Effect of the hydroalcoholic extract of *Heracleum persicum* (Golpar) on Folliculogenesis in female Wistar rats. *Cell J Spring*. 2012;14:47–52. PMC3635820 23626937

[18] Kevenaar EM, Meerasahib FM, Kramer P, et al. Serum anti-mullerian hormone levels reflect the size of the follicle pool in mice. *Endocrinology*. 2006;14:3228–3234. doi:10.1210/en.2005-1588 16556768

[19] Nathalie J, Nathalie D. Anti- mullerian hormone. Encyclopedia of Endocrine Disease; 2017. Available from: http://www.sciencedirect.com/topics/neuroscience/anti-mullerian-hormone. Accessed November 22, 2017.

[20] Motohiro K, Amanda ES, Zhang LH, et al. AMH as a contraceptive that protects the ovarian reserve during chemotherapy; 2016. Available from: http://www.pnas.org/content/114/9/E1688.full.pdf. Accessed November 22, 2017. 10.1073/pnas.1620729114 PMC5338508 28137855

[21] Lamda H, Goldmeier D, Mackie NE, Scullard G. Anti-retroviral therapy is associated with sexual dysfunction and with increased oestradiol levels in men. *Int J STD Aids*. 2004;15:234–237. doi:10.1258/095646204773557749 15075015

[22] Fan MD, Maslow B-S, Santoro N, Schoenbaum E. HIV and the menopause. *Menopause Int*. 2007;14:163–168. doi:10.1258/mi.2008.008027 19037065

[23] Kahwati LC, Haigler L, Rideout S. What is the best way to diagnose menopause? *J Fam Pract*. 2005;54:1000–1002. 16266610

[24] Taylor-Robinson SD. Do medical algorithms always benefit the patient? - A patient perspective. *QJM*. 2023;116:474–475. doi:10.1093/qjmed/hcac254 36355474

